# Congenital malformations at birth in Central India: A rural medical college hospital based data

**DOI:** 10.4103/0971-6866.73412

**Published:** 2010

**Authors:** Amar Taksande, Krishna Vilhekar, Pushpa Chaturvedi, Manish Jain

**Affiliations:** Department of Pediatrics, Mahatma Gandhi Institute of Medical Sciences, Sevagram, Wardha, Maharashtra - 442 102, India

**Keywords:** Cardiovascular, congenital, infant, malformation, musculoskeletal

## Abstract

**OBJECTIVE:**

To study the incidence of congenital anomalies and the associated risk factors in Department of Pediatrics at Mahatma Gandhi Institute of Medical Sciences, Sevagram, Wardha, a rural medical college hospital in central Maharashtra.

**MATERIALS AND METHODS:**

All the intramural deliveries between 1 January 2005 and 31 July 2007 comprised 9386 births and their 9324 mothers (62 mothers gave birth to twin babies). The newborns were examined and assessed systematically for the presence of congenital anomalies, system wise distribution of anomalies and risk factors attributable.

**RESULTS:**

Out of the total 9386 deliveries, 9194 were live births and 192 were stillbirths. The total number of babies with congenital malformations was 179 (1.91%). Out of the 9262 singleton births, 177 (1.05%) were malformed, whereas 2 of the 62 pairs of twins had birth defects. Nine of the 179 malformed babies (5.02%) were still born. Prematurity, increased maternal age, increasing birth order and low birth weight were found to have a higher risk of congenital anomalies. Cardiovascular malformations were most common in live births, followed by musculoskeletal and genitourinary anomalies.

**CONCLUSION:**

Congenital anomalies are a major cause of stillbirths and infant mortality. Evaluation of cardiovascular system to rule out congenital heart disease in high-risk mothers’ babies is the important factor to be considered.

## Introduction

Congenital malformation represents defects in morphogenesis during early fetal life. According to the World Health Organization (WHO) document of 1972, the term congenital malformations should be confined to structural defects at birth.[1] The leading causes of infant morbidity and mortality in poorer countries are malnutrition and infections, whereas in developed countries they are cancer, accidents and congenital malformations. Congenital anomalies account for 8–15% of perinatal deaths and 13–16% of neonatal deaths in India.[2,3] Patients with multiple congenital anomalies present a relatively infrequent but tremendously difficult challenge to the pediatrician. The proportion of perinatal deaths due to congenital malformations is increasing as a result of reduction of mortality due to other causes owing to the improvement in perinatal and neonatal care. In the coming decades, this is going to be a leading cause of morbidity and mortality in centers providing good neonatal care. The present study was carried out with the aim to determine the overall rate of congenital malformations, incidence in live births and stillbirths, as well as incidence affecting various organ systems, at a rural medical college hospital in Maharashtra and compare them to previous studies.

## Materials and Methods

This study was conducted in Department of Pediatrics at Mahatma Gandhi Institute of Medical Sciences, Sevagram, Wardha, a rural medical college hospital in central Maharashtra. All the intramural deliveries between 1 January 2005 and 31 July 2007 comprised the study material. There were a total of 9194 live births and 192 stillbirths during this period. The study material comprised 9386 births (live and still) and their 9324 mothers (62 mothers gave birth to twin babies). All the newborns were looked for congenital malformations soon after birth and everyday during routine ward rounds. Relevant information regarding maternal age, gestational age, sex, community, birth weight, birth order and consanguinity was documented. Significant antenatal history like maternal illness, ingestion of drugs, exposure to radiation and complications of labor was recorded. Antenatal ultrasonography (USG) findings were noted. Relevant radiological, histo-hematological and genetic tests were carried out. Autopsy was done on stillbirth and neonatal death, whenever parents consent could be obtained. Karyotyping was done. A meticulous general and systemic examination was carried out by a consultant at the time of birth to detect any malformations. Ultrasound was employed routinely to detect multiple congenital anomalies and to rule out majority of the internal congenital anomalies. 2D echocardiography was also used for all congenital heart diseases, along with the routine X-ray chest and electrocardiogram. Other investigations were intravenous pyelonephrography and retrograde cystourography. Computed tomography (CT) scan and magnetic resonance imaging (MRI) were advised only for certain special cases and these cases were followed up for a period of 1 year. During the follow-up, the babies were re-examined to detect any other malformations. Malformations were divided into major and minor; major malformation interferes considerably with the function of all or part of the infant, minor malformation gives no serious medical or cosmetic consequences to the patients. The major malformations were divided into central nervous system (CNS), musculoskeletal, gastrointestinal, genitourinary, cardiovascular system (CVS), syndromes and miscellaneous disorders. Statistical analysis was done using Z test and Chi-square test.

## Results

Out of the total 9386 deliveries, 9194 were live births and 192 were stillbirths. The number of babies with congenital malformations diagnosed at birth or within the first week of life was 164, while the total number of malformations was 179 (1.91%).Table 1 gives the frequency and sex distribution of congenital malformations. Out of the 9262 singleton births, 177 (1.05%) were malformed, whereas 2 of the 62 pairs of twins had birth defects. The sexwise distribution was 62% males and 38% females, giving an M:F ratio of 1.63:1, and there were three cases of ambiguous genitalia. Congenital malformations were seen more significantly in stillbirths (*P* < 0.01) as compared to live births, the frequency being 4.68% and 1.84%, respectively. Nine of the 179 malformed babies (5.02%) were still born. Table 2 shows the frequency of congenital malformations in relation to fetal and maternal factors. Women less than 20 years had 1.11% babies with congenital anomalies, and the mothers of babies with congenital anomalies were mostly between 20 and 30 years, i.e. 90.49%, and 8.40% of the mothers were above 30 years.

**Table 1:** Congenital Malformations: Frequency and Sex Distribution

	Total cases	Malformed patients	Percentage
Total births	9386	179	1.90
Still births	192	9	4.68
Live births	9194	170	1.84
Male	4813	121	2.51
Female	4570	58	1.26
Ambiguous	3	0	0

**Table 2:** Frequency of Congenital Malformations in relation to various fetal and maternal factors

	Fetal Factors		Percentage
	Total cases	Malformed patients	
Birth weight (grams)			
<1000	34	2	5.88
1000-1500	188	6	3.19
1501-2000	681	28	4.11
2001-2500	2812	46	1.63
>2501	5671	97	1.71
Gestation			
Preterm	389	20	5.14
Term	8676	156	1.79
Postterm	321	3	0.93
	**Maternal factor**		
Maternal Age (years)			
< 20	561	2	0.35
21-25	6132	130	2.12
26-30	2215	32	1.44
> 30	416	15	3.60
Parity			
Primi	3321	62	1.86
Para 1-3	5768	103	1.78
Para 4 and more	235	14	5.95

History of parental consanguinity was present in 14 cases of congenital malformations in our study. Babies with congenital anomalies were of the first order (34.63%), and second order to third order (51.95%). More than four or fourth birth order was associated with 13.40% of the anomalies. There was a history of oligohydramnios in 13/179 (7.26%) cases and polyhydramnios in 7/179 (3.91%) cases. Also, 19/179 mothers (10.61%) had a history of previous abortions, 6/179 (3.35%) were diabetic mothers and 5/179 (2.79) had a history of congenital heart disease in previous child or malformed babies. Table 3 shows the systemic distribution and the incidence of individual congenital malformations. Cardiovascular malformations were most common in live births, followed by musculoskeletal malformations. The CNS defects were most commonly seen in still born.

**Table 3:** Distribution and incidence of individual congenital malformations

Type of Defect	Total Number	Rate/1000 births
Cardiovascular System		
Acyanotic CHD	23	2.5
Cyanotic CHD	9	0.97
Complex CHD	6	0.65
Central Nervous System		
Microcephaly	3	0.32
Dandy Walker Malformation	3	0.32
Hydrocephalus	2	0.21
Meningoencephalocele	1	0.1
Meningomyelocele	2	0.21
Spina Bifida	2	0.21
Encephalocele	1	0.1
Meningocele	1	0.1
Kidney		
Polycystic Kidney	4	0.43
Hydroureter	2	0.21
Posterior Urethral valve	3	0.32
Genitalia System		
Hypospadias	5	0.54
Micropenis	4	0.43
Ambiguous Genitalia	3	0.32
Congenital Hydrocele	2	0.21
Undescended Testis	5	0.54
Epispadias	3	0.32
Gastrointestinal System		
Diaphrgmatic Hernia	3	0.32
Duodenal Atresia	2	0.21
Omphalocele	1	0.1
Extrophy of bladder	2	0.21
Exomphalos	1	0.1
Imperforated Anus	2	0.21
Gastroschisis	1	0.1
Tracheo-oesophageal fistula	2	0.21
Cleft Lip/ Palate	9	0.97
Musculoskeletal System		
Craniosynostosis	3	0.32
Talipes	11	1.19
Hemimelia	3	0.32
Polydactaly / Syndactyly	15	1.3
Osteogenesis Imperfecta	2	0.21
Hemivertebrae	2	0.21
Syndrome		
TAR syndrome	1	0.1
Arthogyropsis Multiplex Conganita	1	0.1
Pierre Robin Syndrome	1	0.1
Prune Belly Syndrome	1	0.1
Down Syndrome	4	0.43
Respiratory System		
Laryngomalacia	1	0.1
Tracheal Atresia	1	0.1
Skin		
Skin tag over face and hand	4	0.43
Preauricular Tag	6	0.65
Hemangioma	4	0.43
Giant Hairy Naevus	1	0.1
Eye		
Anophthalmia	1	0.1
Microopthalmia	2	0.21
Congenital Ptosis	1	
Defect of Spine		
Sacrococcygeal Sinus	2	0.21
Single Umbilical Artery	12	0.21
Miscellaneous	11	1.3

## Discussion

Congenital anomalies are important causes of still births and infant mortality, and are contributors to childhood morbidity. The number of birth defects in infants is increasing antenatally and during the neonatal period due to advanced diagnostic technology, especially USG. The incidence of congenital malformation in the present study was 1.90%, which is comparable with the earlier studies from the same hospital, which reported an incidence of 2.72 and 1.24% subsequently.[4,5] When autopsies are performed in the hospitals, the incidences of birth defect is increases upto 3times higher. Higher autopsy rates at Chandigarh and Pondicherry centers reported a higher incidence of congenital malformations.[5,6] This study revealed higher incidence of anomalies in stillbirths (4.68%), which is in concordance with a previous report.[4,5] Association of low birth weight with increased risk of congenital malformations was very well documented.[6] Our finding is in accordance with this. The incidence of congenital anomalies was significantly higher in preterm babies as compared to full term babies.[7] Previous studies have reported male preponderance amongst congenital malformed babies,[6] which was statistically insignificant in our study. Previous data showed a definite increase in incidence of congenital malformation in babies born to consanguineous parents.[7] Fourteen cases had a history of consanguinity in our present study. This study has statistically shown that mothers, above 30 years of age, stand at a higher risk of producing malformed babies. Sugunabai[8] reported a higher incidence of malformation in the babies born to mothers aged over 35 years, whereas Datta *et al*.[5] documented statistically insignificant association of increased maternal age and congenital anomalies. Previous studies have[6] reported that significantly higher incidence of malformation among the mothers of gravida 4 or more and our results are consistent with this finding. This indicates that as the birth order increases, the incidence of congenital anomalies also increases. The previous study evaluated the factors that significantly increase the risk of congenital malformations to be presence of hydramnios, maternal febrile illness in the first trimester, past history of abortions, diabetic mother, eclampsia, previous abortion and history of congenital heart disease in previous child or malformed babies. Certain maternal diseases may occasionally lead to increased risk of birth defects. According to Ordonez *et al*.,[9] diabetes mellitus, arterial hypertension, and hypothyroidism show a positive association with congenital malformation. The main aim of the study is to plan measures for maternal and child health, with a main focus on prevention of congenital malformations, by health education, adequate prenatal care and organization of referral networks for major anomalies.

The annual report of Indian Council of Medical Research[10] says that the commonest congenital malformations are cardiac in nature (0.57%). Our study conforms to this. The low prevalence of cardiovascular defects at birth is reported by many studies in the literature, given that their diagnosis is usually made after discharge from the maternity hospital.[11] But in our hospital, regular echocardiography of the child of a mother having a history of high risk pregnancy is done. Kalra *et al*.[12] reported that the CNS defects have the highest incidence, whereas Sugunabi *et al*.[8] reported gastrointestinal malformations to rank the highest. Mathur *et al*.[7] reported that the musculoskeletal abnormalities were the commonest. This study showed that cardiovascular, musculoskeletal and genitourinary were the most commonly affected systems in a descending order of frequency. With regard to the cardiovascular system, ventricular septal defect was the most common lesion found in high-risk mothers who had history of previous child with congenital heart disease, diabetic mothers or those with previous congenital malformed baby. Congenital Talipes Equino varus was the commonest musculocutaneous abnormality observed in our study. Among the genitourinary tract anomalies, hypospadias, undescended testis, and polycystic kidney were the most prevalent lesions. Regarding the central nervous system, the most prevalent anomaly encountered was microcephaly, Dandy walker malformation and meningomyelocele. With special reference to the neural tube defect (NTD), the incidence of NTD has markedly reduced in the developed countries following mass promotion and mandatory prescription of folic acid for pregnant mothers.[13–16] The incidence of congenital heart disease was the leading congenital malformation followed by musculoskeletal system as compare with previous study [Table 4] conducted at MGIMS hospital.

**Table 4:** Comparison of distribution of malformation with previous study

System Involved	Chaturvedi *et al*	Datta *et al*	Present Study
Central Nervous System	15	5	15
Skeletal	22	9	36
Genitourinary	13	3	31
Gastrointestinal	13	8	23
Cardiovascular	6	2	38
Skin	11	0	15
Syndrome	5	3	8
Defect of Spine	2	1	2
Eye	2	4	4
Respiratory Tract	0	0	2
Single Umbilical Artery	0	0	12
Miscellaneous	4	13	11
Total	93	48	197

## Conclusions

Congenital anomalies are a major cause of stillbirths and infant mortality. By thorough clinical examination, the life-threatening congenital malformation must be identified, as early diagnosis and surgical correction of the malformed babies offer the best chance for survival. The incidence of congenital heart disease is much higher than that previously reported from the other parts of the country and from the same area also. Evaluation of cardiovascular system to rule out congenital heart disease in a high-risk mother’s baby is the important factor to be considered.
